# Cannabidiol as a Prophylactic Agent Against Glioblastoma Growth: A Preclinical Investigation

**DOI:** 10.3390/ijms27020757

**Published:** 2026-01-12

**Authors:** Lei P. Wang, Bidhan Bhandari, Sahar Emami Naeini, Breanna Hill, Hannah M. Rogers, Jules Gouron, Nayeli Perez-Morales, Aruba Khan, William Meeks, Ahmed El-Marakby, Nancy Young, Fernando L. Vale, Salman Ali, Gerald Wallace, Jack C. Yu, Ali S. Arbab, Évila Lopes Salles, Babak Baban

**Affiliations:** 1 Georgia Institute of Cannabis Research, Medicinal Cannabis of Georgia LLC., Augusta, GA 30912, USA; semaminaeini@augusta.edu (S.E.N.); harogers@augusta.edu (H.M.R.); gouronjules@gmail.com (J.G.); 2 DCG Center for Excellence in Research, Scholarship, and Innovation (CERSI), Augusta University, Augusta, GA 30912, USA; bibhandari@augusta.edu (B.B.); breannahill2012@gmail.com (B.H.); aelmarakby@augusta.edu (A.E.-M.); nayoung@augusta.edu (N.Y.); esalles@augusta.edu (É.L.S.); 3 Department of Oral Biology, Dental College of Georgia, Augusta University, Augusta, GA 30912, USA; 4 College of Science and Mathematics, Augusta University, Augusta, GA 30912, USA; aperezmorales@augusta.edu (N.P.-M.); arkhan@augusta.edu (A.K.); 5 Medical College of Georgia, Augusta University, Augusta, GA 30912, USA; 6 Department of General Dentistry, Dental College of Georgia, Augusta University, Augusta, GA 30912, USA; 7 Department of Neurosurgery, Medical College of Georgia, Augusta University, Augusta, GA 30912, USA; fvalediaz@augusta.edu (F.L.V.); muhali@augusta.edu (S.A.); 8 Department of Neurology, Medical College of Georgia, Augusta University, Augusta, GA 30912, USA; gewallace@augusta.edu; 9 Department of Surgery, Medical College of Georgia, Augusta University, Augusta, GA 30912, USA; jyu@augusta.edu; 10 Georgia Cancer Center, Medical College of Georgia, Augusta University, Augusta, GA 30912, USA; aarbab@augusta.edu

**Keywords:** cannabidiol, glioblastoma, pretreatment, immune checkpoint, IDO, PD-L1, MGMT, Ki67, tumor microenvironment, neuro-oncology

## Abstract

Glioblastoma (GBM) remains one of the most lethal brain tumors, with current therapies offering limited benefits and high relapse rates. This study presents the first preclinical evidence that pretreatment with inhaled cannabidiol (CBD) before tumor establishment can markedly inhibit GBM progression. We hypothesized that early CBD exposure could prime the immune and molecular landscape to resist tumor growth. C57BL/6 mice were pretreated with inhaled CBD for 3 or 14 days, or with placebo, prior to intracranial implantation of glioblastoma cells. Tumor growth, immune checkpoint expressions (IDO, PD-L1), and key biomarkers (MGMT, Ki67) were analyzed to evaluate tumor dynamics and immune modulation. Fourteen-day CBD pretreatment significantly reduced tumor burden compared with both placebo and 3-day CBD groups, accompanied by decreased IDO, PD-L1, MGMT, and Ki67 expression, which are signatures of a less aggressive tumor phenotype. These findings suggest that prolonged CBD exposure can precondition the tumor microenvironment toward an anti-tumor state, improving disease control and potentially lowering relapse risk. This study introduces a novel concept of CBD pretreatment as an immune-modulatory strategy with high translational potential for glioblastoma management.

## 1. Introduction

The Glioblastoma (GBM) is the most prevalent and aggressive primary malignant brain tumor in adults, characterized by rapid proliferation, extensive infiltration, and a dismal prognosis. Despite decades of research and therapeutic advances, the median survival for GBM patients remains approximately 15 months following diagnosis, with minimal improvement over time [1,2,3,4]. Since 1926, only a limited number of pharmacological agents and a single medical device have received regulatory approval for GBM treatment, underscoring the persistent therapeutic challenges associated with this disease [5]. The etiology of GBM remains poorly understood, and its rising incidence further emphasizes the urgent need for innovative therapeutic and preventive strategies [6].

Emerging research suggests that early intervention or prophylactic approaches may significantly alter the trajectory of GBM progression. Preventative strategies, particularly those targeting molecular pathways involved in tumor initiation and resistance, hold promise for reducing tumor burden, delaying neurological deterioration, and improving overall survival [7,8,9]. Shifting the treatment paradigm from reactive to proactive necessitates a focus on agents capable of modulating GBM-associated pathophysiology before overt tumor development.

Cannabidiol (CBD), a non-psychoactive phytocannabinoid derived from Cannabis sativa, has attracted growing interest for its broad spectrum of therapeutic properties, including anti-cancer and neuroprotective effects [1,3]. Preclinical studies have demonstrated that CBD exerts anti-proliferative, pro-apoptotic, anti-inflammatory, and anti-angiogenic effects across various tumor models, including GBM [3,10,11,12]. Our previous work and other studies have shown that CBD can inhibit GBM growth in the brain by modulating the endocannabinoid system, promoting cell cycle arrest, and impairing angiogenesis [3,10,11,12]. These mechanisms suggest that CBD may be uniquely suited to address both the malignant and neurological aspects of GBM. Moreover, CBD’s established safety profile, lack of psychoactive effects, and reported neuroprotective and anti-inflammatory properties further support its suitability as a preventive agent in at-risk populations.

Notably, the prophylactic potential of inhaled CBD in GBM remains unexplored. Inhalation offers several pharmacokinetic advantages, including rapid systemic absorption, non-invasiveness, and efficient central nervous system delivery via enhanced blood–brain barrier (BBB) penetration [3,10,13]. Furthermore, CBD has been shown to downregulate O6-methylguanine-DNA methyltransferase (MGMT), a DNA repair enzyme associated with resistance to temozolomide, the current standard-of-care chemotherapy for GBM, further highlighting its potential as a sensitizing agent [14,15].

In this study, we investigate the prophylactic potential of chronic CBD administration via inhalation in a murine model of GBM, a novel approach that, to our knowledge, has not been previously explored. While CBD has been studied primarily in the context of treatment following tumor establishment, its utility as a preventive agent represents a significant departure from conventional paradigms focused solely on tumor eradication. The use of inhalation as a delivery method offers distinct translational advantages, including non-invasiveness, rapid systemic uptake, and efficient central nervous system penetration, making it particularly well-suited for potential clinical application in at-risk or pre-symptomatic populations. We hypothesize that sustained pretreatment with inhaled CBD will attenuate tumor growth by inducing apoptosis, suppressing cell proliferation, and downregulating chemoresistance mechanisms such as MGMT expression. Demonstrating the efficacy of this approach could not only establish a foundation for preventive neuro-oncology strategies but also support the clinical development of CBD as a safe, non-toxic, and accessible adjunct to current GBM interventions. If validated, this work would position inhaled CBD as a paradigm-shifting strategy with the potential to delay disease onset, improve treatment responsiveness, and ultimately extend survival in patients with elevated risk for glioblastoma development or recurrence, as well as those in the early stages of GBM.

## 2. Results

### 2.1. Inhaled CBD Pretreatment Inhibits GBM Growth and Improves

#### Tumor-Associated Health Indicators

**Figure 1:**
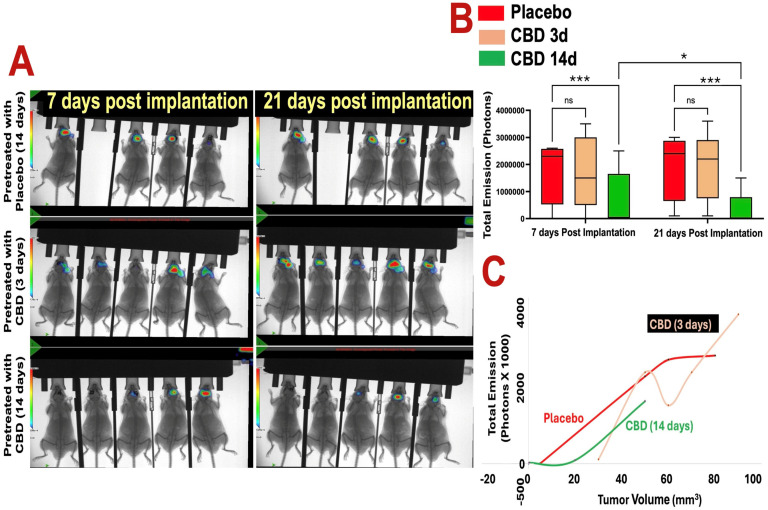
Prophylactic CBD inhalation reduces glioblastoma tumor burden in vivo. (**A**) Optical bioluminescent imaging (BLI) of photon intensities at 7 and 21 days post–tumor implantation shows that 14 days of inhaled CBD pretreatment significantly reduced GBM tumor growth compared to both placebo and 3-day CBD pretreatment groups (*p* < 0.01). Heat map indicates photon emission intensity (scale bar shows photons/sec; blue/green = low signal, yellow/red = high signal). Images show one representative cohort (*n* = 5 mice per group). (**B**) Quantification of total photon emission confirms significant tumor growth inhibition in the 14-day CBD group at both 7 and 21 days post-implantation (* *p* < 0.05 and *** *p* < 0.001, ns = not significant, respectively), while the 3-day pretreatment group did not differ significantly from placebo. (**C**) Cross-validation of BLI signal with tumor volume measurements demonstrates consistent correlation between reduced photon flux and decreased tumor burden in the 14-day CBD group. Each point represents an individual mouse.

In vivo bioluminescent imaging (BLI) demonstrated that 14 days of inhaled CBD pretreatment significantly suppressed GBM tumor growth compared with placebo and 3-day CBD pretreatment at both 7 and 21 days post-implantation (Figure 1A,B). Tumor-associated photon emission was reduced in the 14-day CBD group relative to placebo at day 7 (* *p* < 0.05) and day 21 (*** *p* < 0.001), and relative to the 3-day pretreatment group at day 21 (*** *p* < 0.001). In contrast, the 3-day CBD pretreatment did not significantly differ from placebo. BLI signal strongly correlated with ex vivo tumor volume across all groups (Figure 1C), confirming the reliability of imaging-based tumor assessment.

**Figure 2:**
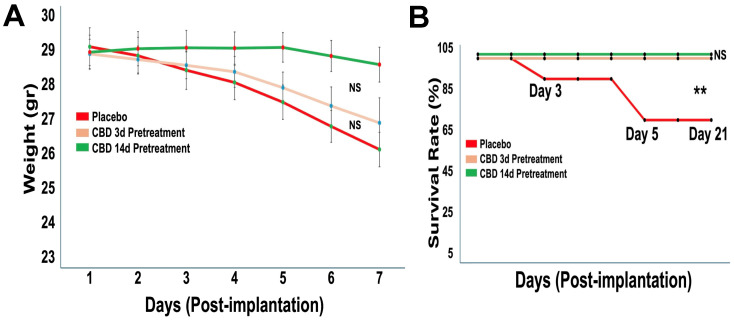
Prophylactic CBD inhalation preserves body weight and significantly improves survival. Mice (*n* = 10 per group, pooled from two independent cohorts) were pretreated with placebo or inhaled CBD prior to tumor implantation. (**A**) Body weight was monitored over 21 days post-implantation. All groups showed modest weight loss over time, with no significant differences between treatment groups. (**B**) Survival was assessed over 21 days post-implantation. Placebo-treated mice exhibited early mortality, whereas both CBD pretreatment regimens (3-day and 14-day) resulted in complete survival throughout the study period (** *p* < 0.05, NS = not significant).

Body weight monitoring over the 21-day period revealed modest weight loss in all groups with no significant differences between treatments (Figure 2A), indicating preserved systemic health. Survival analysis across two independent cohorts showed significantly improved survival in both CBD-pretreated groups compared with placebo (Figure 2B). While placebo-treated mice exhibited 30% mortality by day 5, no deaths occurred in either CBD pretreatment group through day 21 (** *p* < 0.01).

### 2.2. Inhaled CBD Pretreatment Reduces Tumor Volume and Improves

#### Histopathology

**Figure 3:**
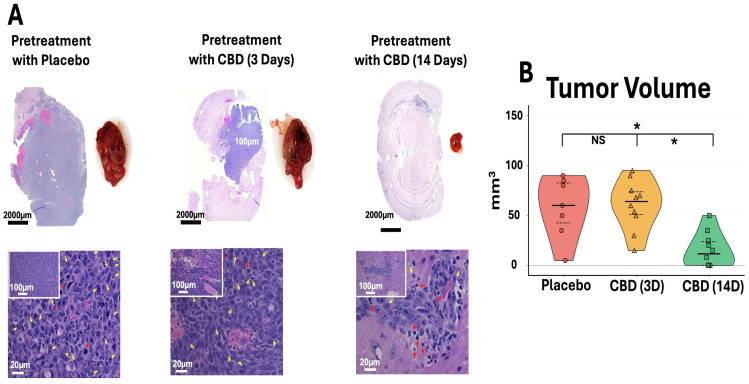
Prophylactic CBD inhalation reduces tumor burden and improves tumor histopathology. (**A**) Representative H&E sections (scale bars: 2000 μm, 100 μm, 20 μm) and gross tumors show reduced size in 14-day CBD group, with decreased mitotic figures (yellow arrows) and apoptotic cells (red arrows). (**B**) Ex vivo tumor volume. Violin plots show individual data (symbols), median (center line), and IQR (dashed lines). 14-day CBD significantly reduced volume versus placebo and 3-day CBD (* *p* < 0.05, NS = not significant). Placebo *n* = 7 (3 died days 3–5, Figure 2B); CBD groups *n* = 10 each. One-way ANOVA with Tukey’s test.

Consistent with the BLI findings, ex vivo brain analysis demonstrated markedly reduced tumor size in the 14-day CBD pretreatment group (Figure 3A). Representative H&E-stained sections and gross tumor specimens showed visibly smaller tumors in mice pretreated with CBD for 14 days compared to placebo and 3-day CBD groups. Histopathological evaluation revealed improved tumor architecture in the 14-day CBD group, with reduced mitotic figures (yellow arrows) and decreased apoptotic cells (red arrows), suggesting that prolonged inhaled CBD pretreatment attenuates both tumor proliferation and cellular stress responses. Quantification of ex vivo tumor volume confirmed significantly reduced tumor burden in the 14-day CBD group compared to both placebo (* *p* < 0.05) and 3-day CBD pretreatment groups (* *p* < 0.05), while 3-day CBD did not differ significantly from placebo (*p* > 0.05) (Figure 3B).

### 2.3. Inhaled CBD Pretreatment Reduces Immune Evasion Gatekeepers in GBM

**Figure 4:**
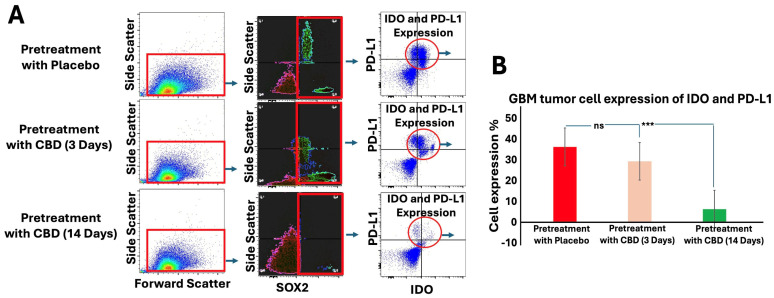
Inhaled CBD Pretreatment Decreases Immune Evasion Signals in GBM. (**A**) Flow cytometry analysis was used to assess the expression of immune evasion signals, IDO and PD-L1, in GBM tumors following 14 days of inhaled CBD pretreatment. Tumor cells were identified based on forward light scatter (FSC) and side light scatter (SSC) (left panels), followed by gating for SOX2 expression, a marker of stem-like properties (middle panels). Dot plots show the reduction of IDO and PD-L1 expression specifically in SOX2-positive cells following 14-day CBD pretreatment. (**B**) Quantification of IDO and PD-L1 expression in SOX2-positive cells revealed a significant decrease in both markers after 14-day CBD pretreatment (*** *p* < 0.001, ns = not significant), compared to the placebo and 3-day CBD groups. These results suggest that prolonged CBD pretreatment reduces immune evasion signals in GBM, particularly in stem-like tumor cells, potentially enhancing anti-tumor immunity.

Mice pretreated with inhaled CBD for 14 days showed a significant reduction in the expression of immune evasion markers, IDO and PD-L1, in GBM tumors compared to both the placebo and 3-day CBD groups. Flow cytometry analysis (Figure 4A) identified tumor cells based on forward light scatter (FSC) and side light scatter (SSC) (left panels), followed by gating for SOX2 expression, a marker of stem-like properties (middle panels). Importantly, 14-day CBD pretreatment not only decreased overall SOX2 expression in the tumors but also specifically reduced IDO and PD-L1 expression within SOX2-positive cells (dot plots). Quantification of these markers in SOX2-positive cells (Figure 4B) revealed a significant decrease in both IDO and PD-L1 expression following 14-day CBD pretreatment (*p* < 0.001). These results suggest that prolonged CBD pretreatment may disrupt immune evasion mechanisms in GBM, particularly in stem-like cells, potentially enhancing anti-tumor immune responses.

### 2.4. Inhaled CBD Pretreatment Reduced MGMT and Ki-67 Expression in GBM

#### Tumors

**Figure 5:**
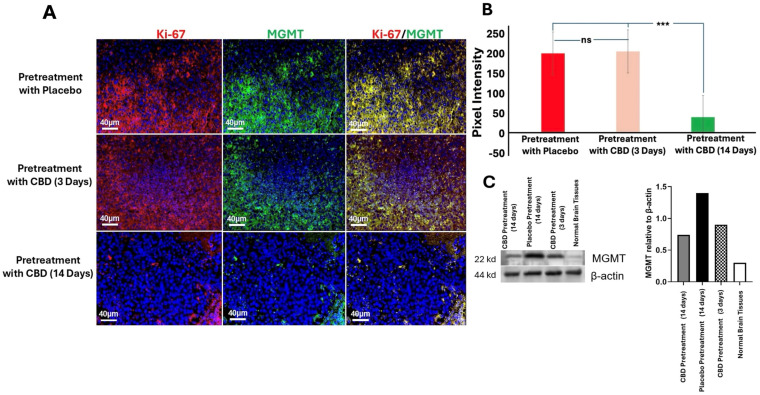
Inhaled CBD Pretreatment Reduces MGMT and Ki-67 in GBM Tumors. (**A**) Immunofluorescence staining of MGMT and Ki-67 in GBM tumors from mice pretreated with inhaled CBD for 14 days, 3 days, or placebo. Representative images show that MGMT and Ki-67 levels were significantly reduced in the 14-day CBD pretreatment group compared to both the 3-day CBD and placebo groups (Scale bar: 40 μm). (**B**) Quantification of staining intensity revealed significantly lower levels of MGMT and Ki-67 in the 14-day CBD pretreatment group compared to the 3-day CBD and placebo groups (*** *p* < 0.001, ns = not significant). These results suggest that CBD pretreatment reduces key biomarkers associated with chemotherapy resistance and tumor proliferation, potentially enhancing therapeutic sensitivity. (**C**) To further validate the effect of CBD on tumor progression, we assessed MGMT expression levels using Western blotting. The blot shows increased MGMT expression in the GBM tumors of mice pretreated with placebo (lane 2) compared to the normal brain tissue used as a negative control (lane 4). Long-term CBD pretreatment (14 days, lane 1) significantly reduced MGMT expression in the tumor compared to acute CBD pretreatment (3 days, lane 3), corroborating the reduction observed in the immunofluorescence analysis.

Immunofluorescence staining and quantification revealed that mice pretreated with inhaled CBD for 14 days exhibited significantly reduced expression of MGMT and Ki-67 compared to both the 3-day CBD and placebo groups (*p* < 0.001) (Figure 5A,B). MGMT is a DNA repair enzyme associated with resistance to alkylating chemotherapy agents, such as Temozolomide (TMZ), and elevated levels are typically indicative of poor therapeutic response [16,17]. Ki-67, a marker of cellular proliferation, is strongly correlated with tumor cell growth, aggressiveness, and poor prognosis [18]. These findings suggest that CBD pretreatment may modulate key biomarkers involved in GBM progression, potentially enhancing the tumor’s sensitivity to treatment and improving therapeutic outcomes. To further confirm these results, we performed Western blotting analysis for MGMT. The Western blot data corroborated the immunofluorescence results, showing a consistent reduction in MGMT protein expression in the 14-day CBD pretreatment group compared to the placebo and 3-day CBD groups (Figure 5C). This additional analysis reinforces the notion that prolonged CBD exposure effectively modulates key biomarkers associated with GBM progression, supporting the potential for CBD to enhance therapeutic efficacy in GBM treatment.

## 3. Discussion

This study demonstrates that prolonged pretreatment with inhaled CBD (14 days) significantly inhibits glioblastoma (GBM) tumor growth in a murine model, whereas shorter pretreatment durations (3 days) or placebo treatments failed to show the same effect. Several key findings support this observation: (1) a notable reduction in tumor volume in the 14-day CBD pretreatment group compared to the placebo and 3-day CBD groups; (2) a decrease in the expression of SOX2, a marker of stem-like properties, along with immune checkpoint regulators IDO and PD-L1 in the 14-day CBD pretreatment group; (3) a reduction in MGMT and Ki-67, which are biomarkers associated with DNA repair and cellular proliferation, respectively.

Importantly, the consistency between bioluminescent imaging and anatomical tumor quantification underscores the robustness of the anti-tumor effect observed with prolonged CBD pretreatment. While individual variability in photon flux patterns was noted—such as flat or modest BLI increases in some mice—this did not preclude a clear histological and volumetric confirmation of tumor suppression. These findings emphasize the need to interpret BLI data in the context of anatomical validation. Moreover, the improved survival seen in the prolonged CBD group further supports the biological relevance of these tumor growth differences. Although detailed imaging and molecular analyses were derived from a representative cohort, this approach ensured consistency in longitudinal data collection and was supported by survival trends replicated across independent experimental cohorts.

The most striking outcome of these findings is the inhibition of GBM growth following prolonged CBD pretreatment, which holds promise not only as a potential therapeutic strategy but also in slowing tumor progression, a critical challenge in GBM treatment [19]. CBD’s early intervention in modulating the tumor microenvironment and molecular pathways could help reduce the tumor cells’ ability to evade immune surveillance, suppress tumor proliferation, and overcome resistance mechanisms. This is particularly significant post-surgery, where CBD pretreatment could help mitigate tumor resistance to therapy, enhance tumor control, and reduce the risk of recurrence. Thus, CBD pretreatment has the potential to improve both survival rates and the overall quality of life for patients, particularly by reducing recurrence and improving chemotherapy efficacy through the regulation of resistance mechanisms like MGMT.

CBD’s favorable safety profile and lack of observed adverse effects—including weight loss, respiratory irritation, or behavioral changes during the 14-day aerosol exposure—further support its candidacy as a prophylactic agent. Preclinical studies have shown that CBD is well-tolerated, with minimal toxicity and no psychoactive effects, even at relatively high doses. Additionally, its ability to modulate oxidative stress, reduce neuroinflammation, and protect neural integrity aligns with its potential preventive role in neurological malignancies like GBM. These attributes offer a strong translational rationale for early administration in high-risk or pre-symptomatic settings, where safety and tolerability are paramount.

The inhalation route of CBD administration offers unique advantages, especially in the context of GBM treatment. While cannabinoids have primarily been investigated through oral or systemic routes, inhalation ensures rapid absorption, improved bioavailability, and enhanced blood–brain barrier (BBB) penetration, making it an ideal non-invasive approach for targeting brain tumors [20,21,22]. Although direct comparative data across routes are limited in GBM, prior studies suggest inhaled CBD outperforms oral forms in other neurological contexts [23]. This evidence supports our rationale for selecting the inhalation route, as briefly discussed earlier in the study. Furthermore, the duration-dependent effect observed in this study highlights the necessity of prolonged CBD exposure to achieve therapeutic effects. The results suggest that 14 days of pretreatment are crucial for modulating key molecular pathways involved in tumor growth and survival, marking a significant finding for optimizing treatment strategies.

The regulation of SOX2-positive cells is another critical outcome of this study. SOX2-positive cells are known for their stem-like properties and play a significant role in tumor resistance and recurrence in GBM [24]. By reducing the expression of immune checkpoint molecules such as IDO and PD-L1 in these cells, CBD pretreatment directly targets the most aggressive and therapy-resistant subpopulation of GBM cells. IDO and PD-L1 are essential for immune evasion and immune suppression, and downregulating these markers could enhance the immune system’s ability to recognize and attack tumor cells, thereby improving treatment efficacy [25,26]. Additionally, the regulation of MGMT and Ki67 further reinforces the potential of CBD as a therapeutic strategy. MGMT, known to confer resistance to TMZ, was significantly reduced following CBD pretreatment, suggesting that CBD could modify GBM tumors’ resistance to TMZ, improving its chemotherapeutic efficacy [16]. Similarly, Ki67, a key marker of cell proliferation, was downregulated by CBD, suggesting that it may reduce the tumor’s proliferative capacity [18]. Prior research has demonstrated that CBD can downregulate MGMT expression in glioma cells, particularly in vitro, through mechanisms involving oxidative stress and modulation of epigenetic pathways [15,27]. These findings provide a rationale for our current hypothesis that similar regulatory mechanisms may underlie the observed MGMT suppression in vivo following inhaled CBD pretreatment.

The CBD-induced reduction of these pivotal biomolecules including MGMT, IDO, PD-L1 and Ki67 may be well due to counter inflammatory and regulatory nature of CBD. CBD is reported to downregulate NF-κB and STAT3 signaling pathways which play central roles in the inflammatory process, immune checkpoint expression, and oncogenic transcription [28]. Additionally, CBD influences endoplasmic reticulum stress and oxidative stress responses in tumor cells, which may suppress tumor-promoting cytokine production and cell cycle progression. Altogether, these interactions may provide reasonable hypothetical rational to explanation the impact of CBD-pretreatment on the immune evasion, proliferation, and DNA repair markers in this mouse model of GBM. Together with the reduction in PD-L1 and IDO in SOX2-positive cells, these results highlight CBD’s multifaceted approach in targeting immune evasion, tumor growth, and treatment resistance, offering a comprehensive strategy to enhance the efficacy of existing therapies and improve long-term outcomes for GBM patients.

This study opens new avenues for research into pretreatment strategies for GBM, a largely underexplored area. The success of inhaled CBD in this model lays the foundation for further exploration of other cannabinoids or related compounds that may exhibit similar anti-tumor effects. Future directions should include validating behavioral tolerance, stress responses, and cognitive outcomes following prolonged CBD aerosol exposure to strengthen translational relevance. Additionally, evaluating the optimal dosing and duration of CBD pretreatment will be crucial in maximizing therapeutic efficacy and minimizing potential side effects. Combining CBD pretreatment with chemotherapy or immunotherapy could also be explored as a synergistic approach to treating glioblastoma.

### Limitations

While our findings are promising, several limitations should be acknowledged. This study was conducted using the GL261 murine glioblastoma model in an immunocompetent host, which is well suited for evaluating neuroinflammatory and immunomodulatory effects but may not fully capture the genetic and phenotypic heterogeneity of human glioblastoma, underscoring the need for validation in additional human-relevant models and clinical trials. The molecular mechanisms underlying CBD’s anti-tumor effects remain incompletely understood, and future studies should further investigate its interactions with cannabinoid receptors, oxidative stress pathways, and epigenetic modulators. Optimal dosing and duration of CBD pretreatment also require further refinement to maximize therapeutic benefit while minimizing side effects.

Although a standardized broad-spectrum CBD formulation was used throughout the study to ensure internal reproducibility, the absence of detailed compositional profiling of minor constituents may limit cross-study comparisons with other CBD preparations. While pharmacokinetics and bioavailability of inhaled CBD were briefly addressed in the Methods section, the lack of direct comparisons across delivery routes within this GBM model limits interpretation; ongoing work in our laboratory aims to address this gap. Although bioluminescent imaging and related outcomes were derived from a representative cohort selected to permit consistent imaging, survival analysis was performed across two independent cohorts to improve statistical power. Finally, while group sizes (*n* = 5 per group, replicated across two cohorts) showed consistent trends, larger-scale studies will be necessary to further validate and generalize these findings.

## 4. Materials and Methods

### 4.1. Animals

Wild-type C57BL/6 (total of 30 mice from 2 independent cohorts, *n* = 5 for each experimental group), 12 week old male mice (obtained from Jackson Laboratories, Bar Harbor, ME, USA) were used to generate the orthotopic GBM model. The animals were housed in the laboratory animal facilities of the Augusta University with free access to food and water. All experiments were conducted under the approval of the Augusta University Animal Care and Use Committee (Protocol # 2011-0062).

### 4.2. Metered Aerosolized Cannabinoid Delivery Device

The metered dose tincture inhaler used in this study (ApelinDx) was generously supplied by Thriftmaster Global Bioscience, Dallas, TX, USA, and has been utilized previously by our group for preclinical evaluation of inhaled cannabidiol (CBD)–based interventions [3]. The formulation consists of a standardized broad-spectrum CBD extract derived from winterized hemp, in which cannabidiol is the predominant cannabinoid, along with trace levels of naturally occurring minor cannabinoids and terpenes typical of broad-spectrum hemp extracts, while remaining compliant with non-psychoactive specifications. Manufacturer-provided documentation indicated consistent batch composition and compliance with non-psychoactive standards.

Each ApelinDx unit contained a total of 1000 mg of formulation, comprising 985 mg of broad-spectrum CBD and 15 mg of co-solvent, surfactant, and propellant. The device delivered approximately 5 mg of CBD per actuation at a controlled flow rate of 200 mL/min. The placebo formulation was identical in composition, except that the active CBD component was replaced with hemp seed oil.

For adaptation to the murine model, the inhaler was modified by incorporating an additional nozzle component to improve regulation of inhalation volume and delivery consistency. While comprehensive cannabinoid compositional profiling and formal pharmacokinetic characterization of this specific formulation were not performed as part of the present study, the delivery platform and inhalation route have been employed by our group in prior preclinical investigations to assess biological and therapeutic outcomes [3]. In addition, the pharmacokinetic properties of inhaled CBD, including rapid systemic absorption and improved bioavailability relative to oral administration, have been described previously in independent studies [29,30].

To minimize variability in aerosol exposure, inhaled delivery was standardized using a calibrated actuator and controlled environmental conditions within the exposure chamber. All animals received inhaled doses from freshly primed canisters, ensuring consistency across treatment sessions. Although minor variability is inherent to aerosolized delivery systems, these procedures were designed to minimize fluctuations in exposure across animals and experimental sessions.

### 4.3. Pre-Treatment with CBD Inhalation

Mice were randomly allocated using simple randomization into three experimental groups (*n* = 5/group): one control group and two CBD-treated groups. All mice were selected within a narrow weight range (28–29.5 g) and weighed prior to treatment initiation to ensure consistent body-weight-adjusted dosing (based on average weight of 29 g). The CBD groups received six actuations of CBD (approximately 10 mg per animal) daily, starting on day two weeks (day-14) and three days (day-3) prior to tumor implantation in the brain. To minimize handling stress, mice were allowed to acclimate before CBD inhalation, with appropriate time intervals between the six actuations. These two distinct pre-treatment timeframes were chosen to assess the time-dependent effects of CBD on tumor progression. The dose, as used in our previous work, was calculated based on effectiveness and tolerability of CBD to achieve antitumor effect [3]. The control group was administered a placebo using a calibrated inhaler. As described previously [3], the inhalation procedure was conducted in a controlled environment to ensure accurate dosing and minimize variability. This pre-treatment protocol was designed to investigate the potential impact of CBD on modulating the tumor microenvironment and inhibiting tumor growth in the brain.

### 4.4. Tumor Cell Preparation and Orthotopic Glioblastoma Model in Mice

To establish the orthotopic glioblastoma (GBM) model, we followed previously validated protocols [3]. Luciferase-expressing GL261 murine glioma cells, which are syngeneic to C57BL/6 mice, were cultured in RPMI-1640 medium supplemented with 10% fetal bovine serum under standard conditions. On the day of implantation, cells were harvested and suspended in serum-free medium.

Mice were anesthetized with 3% isoflurane for induction and maintained at 1.5–2% throughout the surgical procedure. Following sterile preparation, a cranial burr hole was carefully drilled 2.25 mm lateral and 1 mm posterior to the bregma, ensuring the dura remained intact. A total of 30,000 GL261 cells suspended in 3 μL of media were loaded into a 10 μL Hamilton syringe fitted with a 26-gauge needle. The needle was inserted to a depth of 4 mm and then retracted to 3 mm, where the injection was performed. To ensure consistent and accurate delivery, all injections were performed using a motorized injector system, which allows for highly precise and reproducible administration of cells at the targeted depth and location. This standardized approach minimizes variability across experimental groups. To minimize cell reflux, the needle was withdrawn incrementally in 1 mm steps, beginning 2–3 min after cell delivery. The injection site was sealed with bone wax, and the exposed skull was disinfected using Betadine prior to skin closure with sutures. Postoperative pain management was provided via a single subcutaneous dose of buprenorphine (1 mg/kg). Tumor development was monitored on day 8 post-implantation using in vivo bioluminescence imaging. Mice received an intraperitoneal injection of D-luciferin (100 μL at 150 mg/kg), and bioluminescent signals were captured using the AmiX optical imaging system (Spectral Instruments Imaging, Tucson, AZ, USA). Photon emission (photons/sec/mm^2^) was quantified using Aura imaging software (version 4.0.0; Spectral Instruments Imaging, LLC), enabling visualization of primary tumor burden and potential metastatic spread.

### 4.5. Bioluminescence Imaging for Tumor Monitoring

To assess tumor burden and progression, bioluminescence imaging was performed on day 8 following intracranial tumor cell implantation. Mice received an intraperitoneal injection of D-luciferin (100 µL at a dose of 150 mg/kg), after which in vivo optical images were captured to evaluate both primary tumor growth and potential metastatic dissemination. Imaging was conducted using the AmiX optical imaging platform (Spectral Instruments Imaging, Tucson, AZ, USA), and signal intensity was measured in photons per square millimeter per second. Quantitative analysis of the emitted light was carried out using Aura Imaging Software (version 4.0.0; Spectral Instruments Imaging, LLC).

To longitudinally monitor tumor development, additional imaging sessions were conducted on days 7 and 21 post-implantation. At the end of the imaging schedule, all animals were humanely euthanized, and brain tissues were harvested for downstream applications, including histopathological evaluation, immunofluorescence staining, and flow cytometric analyses.

### 4.6. Visualization of Glioblastoma Lesions

To enable macroscopic examination of intracranial tumor growth, a craniotomy was performed on mice to expose the tumor-bearing region of the brain. Upon surgical exposure, high-resolution digital images were acquired to capture the gross morphology and visual characteristics of the tumor. This imaging approach facilitated direct comparison of tumor presentation between the CBD-pretreated and placebo-treated groups, offering real-time visual confirmation of differential tumor progression within the brain tissue.

### 4.7. Histologic Analysis of Tumor Tissue

Histopathological analysis was performed following previously established protocols [1,3]. Glioblastoma tissues were freshly excised and fixed in 10% neutral buffered formalin (HT50-1-128; Sigma-Aldrich, St. Louis, MO, USA). Samples were then processed through standard dehydration steps and embedded in paraffin. All procedures were carried out at ambient room temperature. Paraffin-embedded brain sections were cut into 4 μm slices and stained with hematoxylin and eosin (H&E) using conventional histological techniques. Tissue morphology and tumor architecture were examined under a Zeiss brightfield microscope (Zeiss USA, White Plains, NY, USA).

### 4.8. Fluorescence-Based Immunohistochemical Analysis

Immunofluorescence staining was carried out on paraffin-embedded brain tumor sections using previously established protocols [3]. Tissue sections were incubated with fluorescently labeled primary antibodies targeting MGMT O6-methylguanine-DNA methyltransferase; Novus Biologicals (Centennial, CO, USA, Cat# NB100-168SS) and the proliferation marker Ki-67 (Thermo Fisher Scientific, Waltham, MA, USA, Cat# 12-5698-82). Nuclear counterstaining was performed with DAPI (4′,6-diamidino-2-phenylindole) to facilitate cellular visualization.

Fluorescent images were acquired using a Zeiss fluorescence microscope (Zeiss USA, White Plains, NY, USA). Quantitative image analysis was performed by selecting regions of interest (ROIs) using the lasso tool in Adobe Photoshop CS4 Extended (version 11.0; Adobe Systems, San Jose, CA, USA). Within these ROIs, integrated density (pixel intensity) and mean gray value (a measure of fluorescence brightness) were recorded to assess relative marker expression levels.

### 4.9. Flow Cytometric Analysis

For flow cytometry, a single-cell suspension was prepared from GBM tumor tissue by passing the sample through a 100 μm cell strainer (BD Biosciences, San Diego, CA, USA), followed by centrifugation at 252× *g* for 10 min. The resulting cell pellet was then subjected to a standard flow cytometry staining protocol as previously outlined [3]. Cells were fixed, permeabilized, and subsequently stained for the detection of intracellular signaling markers. Specific antibodies used included anti-SOX2 (APC-conjugated anti-mouse SOX2, R&D Systems, Minneapolis, MN, USA, Cat# IC2018A), anti-PD-L1 (Alexa Fluor 488-conjugated anti-mouse PD-L1, Thermo Fisher Scientific, Cat# 53-5982-82), and anti-IDO (PerCP-conjugated anti-mouse IDO, Thermo Fisher Scientific, Cat# 46-9473-82). The stained cells were analyzed using a NovoCyte Quanteon flow cytometer (Agilent Technologies, Santa Clara, CA, USA), with data analysis performed using FlowJo V10 software. To validate antibody specificity and exclude potential non-specific binding to Fc receptors or other cellular components, appropriate isotype controls were included in all experiments. These isotype controls matched the primary antibody in terms of host species, isotype, and conjugation type, ensuring accurate results.

### 4.10. Western Blotting

Western blotting was used to assess the expression levels of MGMT in tumor tissues [31]. The samples were homogenized in RIPA lysis buffer in presence of protease inhibitor (Thermo Scientific, Waltham, MA, USA). Protein concentration was determined using the BCA assay. Homogenates (50 μg protein) were separated by electrophoresis on a 4–15% precast polyacrylamide gel (Bio-Rad, Hercules, CA, USA) and transferred to PVDF membrane that was previously soaked in methanol using PowerPac™ Universal power supply and Trans-Blot Turbo transferring system (Bio-Rad Laboratories, Inc., Hercules, CA, USA), respectively. Membranes were probed simultaneously with anti-mouse MGMT monoclonal antibody (O6-methylguanine-DNA methyltransferase, Novus Biologicals, USA, Cat# NB100-168SS) and β-actin antibody (Thermo Fisher, Waltham, MA, USA) for 24 h. Proteins were detected using appropriate secondary antibodies conjugated with different fluorescent dyes compatible with the Odyssey imaging system, allowing simultaneous detection without stripping. Membranes were stripped and re-probed for β-actin (Thermo Fisher, Waltham, MA, USA) to demonstrate equal loading. The reactive bands were visualized using the Li-Cor Odyssey FC system. The results were quantified by densitometry analysis utilizing ImageJ NIH software (version 1.54r; National Institutes of Health, Bethesda, MD, USA) and expression levels were reported relative to β-actin.

### 4.11. Statistical Analysis

Data are presented as mean ± SEM unless otherwise indicated. Sample sizes (*n* = 10 mice per group, pooled from two independent cohorts) were determined by power analysis to detect a 30% difference in tumor burden with 80% power at α = 0.05. Tumor burden (bioluminescence and tumor volume) was analyzed using two-way ANOVA with Tukey’s post hoc test. Body weight data were analyzed by repeated-measures two-way ANOVA. Survival was assessed using Kaplan–Meier analysis with log-rank (Mantel–Cox) testing. Correlations between bioluminescent signal and tumor volume were evaluated by linear regression (R^2^). Statistical significance was defined as * *p* < 0.05, ** *p* < 0.01, *** *p* < 0.001. All analyses were performed using GraphPad Prism 9.0 (GraphPad Software).

## 5. Conclusions

This preclinical investigation demonstrates that prolonged inhaled CBD pretreatment significantly suppresses glioblastoma (GBM) progression in a murine model by targeting multiple hallmarks of tumor biology. Key outcomes include reduced tumor burden and modulation of molecular markers associated with tumor stemness (SOX2), immune evasion (PD-L1, IDO), cellular proliferation (Ki67), and chemoresistance (MGMT). These effects were accompanied by immunomodulatory and antiproliferative shifts in the tumor microenvironment, consistent with a transition toward a less aggressive phenotype. Notably, the superiority of a 14-day pretreatment regimen over shorter durations underscores the importance of sustained exposure and supports inhalation as a promising delivery route for central nervous system therapeutics. Collectively, these findings support the development of CBD as a non-invasive, prophylactic adjunct to standard GBM treatments and provide a strong rationale for further translational studies aimed at optimizing CBD-based interventions to improve clinical outcomes in this aggressive malignancy.

## Data Availability

The original contributions presented in this study are included in the article. Further inquiries can be directed to the corresponding authors.

## Abbreviations

The following abbreviations are used in this manuscript:

GBM	Glioblastoma
CBD	Cannabidiol
MGMT	O6-methylguanine-DNA methyltransferase
IDO	indoleamine 2,3-dioxygenase
PD-L1	Programmed cell death ligand 1
FSC	Forward Scatter
SSC	Side Scatter
